# A Case Report on Premature Twins: Primary Congenital Glaucoma or Large Cupping Disks Mimicking Primary Congenital Glaucoma?

**DOI:** 10.7759/cureus.17108

**Published:** 2021-08-11

**Authors:** Shunsuke Nakakura, Etsuko Terao, Nanami Kuroda, Shota Fujio, Yuka Hirose, Akio Tabuchi, Yoshiaki Kiuchi

**Affiliations:** 1 Ophthalmology, Saneikai Tsukazaki Hospital, Himeji, JPN; 2 Ophthalmology, Hiroshima University, Hiroshima, JPN

**Keywords:** primary congenital glaucoma, child glaucoma, preterm, twins, genetics, intraocular pressure

## Abstract

Large cupping of the optic disk in a baby or a child can be indicative of primary congenital glaucoma. Primary congenital glaucoma is often refractory to treatment, and lifelong management and follow-up are necessary; therefore, diagnosis requires careful consideration. In this study, we describe the case of twins in whom primary congenital glaucoma was initially suspected due to large cupping of the optic disks. Twin babies (a boy and a girl weighing 455 g and 592 g respectively), born prematurely (at 28 gestational weeks), were referred to our hospital eight months after birth because large cupping of the optic disks was detected during follow-up for retinopathy of prematurity. According to color fundus photographs, the cup/disk ratios in both eyes of both babies ranged from 0.75 to 0.86. However, the axial length ranged from 18.57 to 19.91 mm, the anterior chamber depth ranged from 2.68 to 2.93 mm, and the horizontal diameters of the corneas, which were clear, ranged from 10 to 10.5 mm. The intraocular pressures (IOPs), as measured by a rebound tonometer, were 15.3-19.7 mmHg. Glaucoma was strongly suspected due to the large cupping of the optics disks; however, other ocular biometric tests demonstrated that the eyes were normal. After eight months of follow-up without any medication or intervention, the IOPs stabilized between 6-22.1 mmHg, the refractive errors were between -2.5 and 0 diopters, and we found no apparent enlargement of the optic disks. In addition, we investigated both parents’ optic disks to evaluate the genetic factors and found that they had relatively large C/D ratios (0.68-0.79). These premature twins exhibited glaucoma-like optic disks with large cupping, but no solid glaucomatous changes were observed with ocular biometry and IOP testing. We concluded, therefore, that the early birth and lower birth weights may have been associated with the large cupping of the patients’ optic disks. To differentiate between normal physiological cupping and primary congenital glaucoma, ocular morphological examination of the eye, IOP measurements, and investigation of the parents’ optic disks were useful.

## Introduction

Primary congenital glaucoma (PCG) is a rare variety of glaucoma and commonly presents between the ages of three to nine months; however, the most severe form is newborn-onset [1]. Elevated intraocular pressure (IOP) is associated with the classic “triad” of symptoms (photophobia, epiphora, and blepharospasm) which occurs due to rapid expansion of the eye, causing buphthalmos, corneal enlargement, Haab striae, and subsequent corneal edema and opacification [1]. If the IOP increases consistently, optic cup enlargement will progress.

In this study, we describe the case of twins in whom PCG was initially suspected due to large cupping of the optic disks in both eyes of both children. In this case report, we describe how to distinguish between PCG and normal physiological cupping.

## Case presentation

This non-interventional, retrospective study received approval from the Institutional Review Board of Saneikai Tsukazaki Hospital (No. 211019) and was performed according to the Declaration of Helsinki.

Premature (born at 28 gestational weeks) twins (a 455-g boy and a 592-g girl) were referred to our hospital eight months after birth because large cupping of the optic disks was found during follow-up for retinopathy of prematurity. During their first visit to our hospital, we investigated the babies carefully without sedatives. They had no signs of the classic “triad” of symptoms of PCG (photophobia, epiphora, and blepharospasm). In the boy, hand-slit lamp examination showed no apparent Haab’s striae, no corneal edema, no conjunctival injection, and normal anterior chamber depth (i.e., slightly shallower than that of adults). His horizontal corneal diameters were 10 mm in the right eye and 10.5 mm in the left eye. IOP, measured by an IcarePRO rebound tonometer (Icare Finland Oy, Helsinki, Finland), was 19.7 mmHg in the right eye and 16.9 mmHg in the left. Axial length and anterior chamber depth, measured by ultrasound (UD-8000AB, Tomey, Nagoya, Japan), were 19.91/2.73 mm in the right eye and 19.52/2.90 mm in the left eye. In the girl, hand-slit lamp examination also revealed no apparent signs of PCG. Her horizontal corneal diameters were 10.5 mm in the right eye and 10 mm in the left eye. IOP was 18.9 mmHg in the right eye and 15.3 mmHg in the left. The axial length and anterior chamber depth were 18.57/2.93 mm in the right eye and 18.81/2.68 mm in the left.

**Figure 1:**
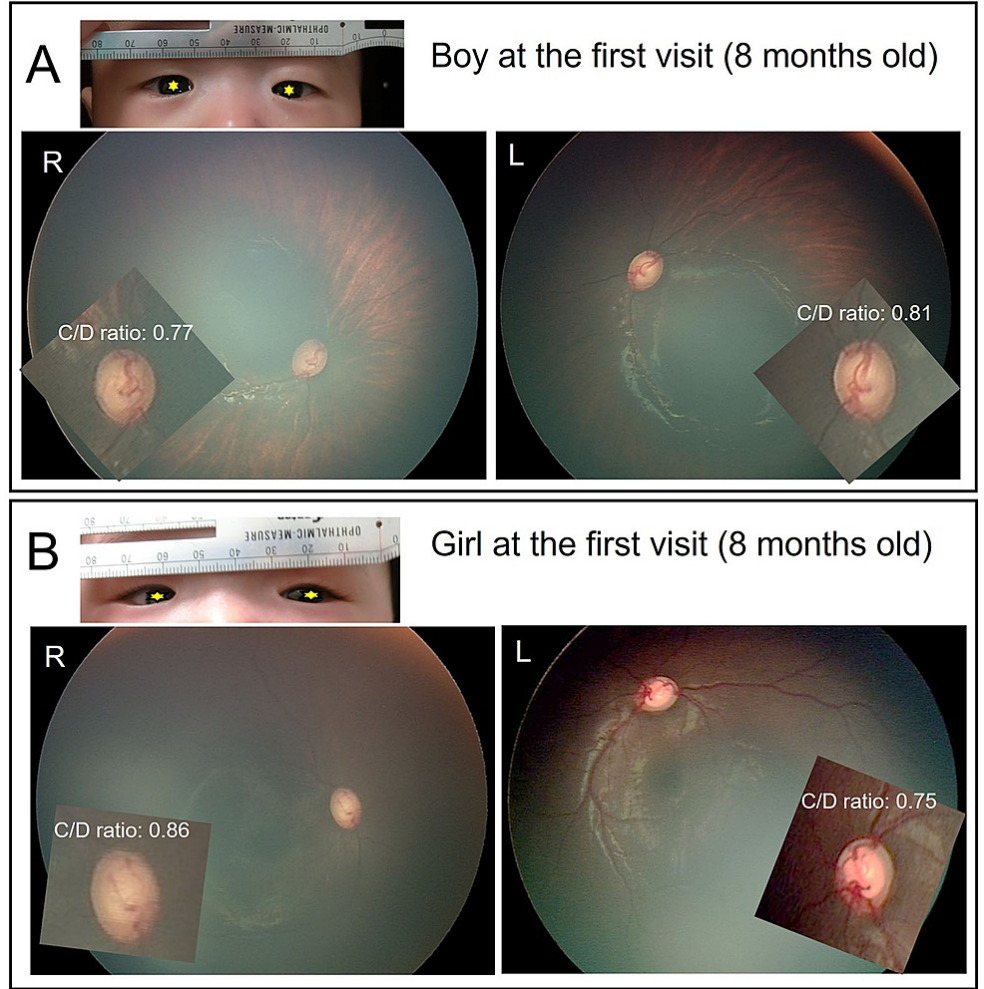
Color fundus photography and facial photographs at the first visit (eight months of age)

After obtaining the ocular biometry measurements, we took color fundus photographs using a wide-field digital camera (RetCam 3, Natus, Pleasanton, California, USA) while the patients’ pupils were dilated. Figure 1 shows the color fundus photographs with magnified optic disk and facial photographs. In the boy patient (Figure 1A), the vertical and horizontal cup/disk ratio (C/D ratio) was 0.77/0.76 in the right eye and 0.81/0.76 in the left eye. In the girl patient (Figure 1B), the vertical and horizontal C/D ratio was 0.86/0.78 in the right eye and 0.75/0.83 in the left eye.

For their first examination during their first visit, the ocular biometry and IOP results were normal for babies of their age. However, the fundus color photographs showed large cupping of the optic disks (vertical C/D ratios ranged from 0.75 to 0.86), strongly suggesting glaucoma. If PCG is suspected, it may be desirable to perform an examination under general anesthesia. However, we could, fortunately, measure IOP without crying using a rebound tonometer, and obtain almost the necessary information except for central corneal thickness and gonioscopic photography. We consulted specialists in child glaucoma (authors Y.K. and A.T.) and followed up carefully without any drugs or intervention.

**Figure 2:**
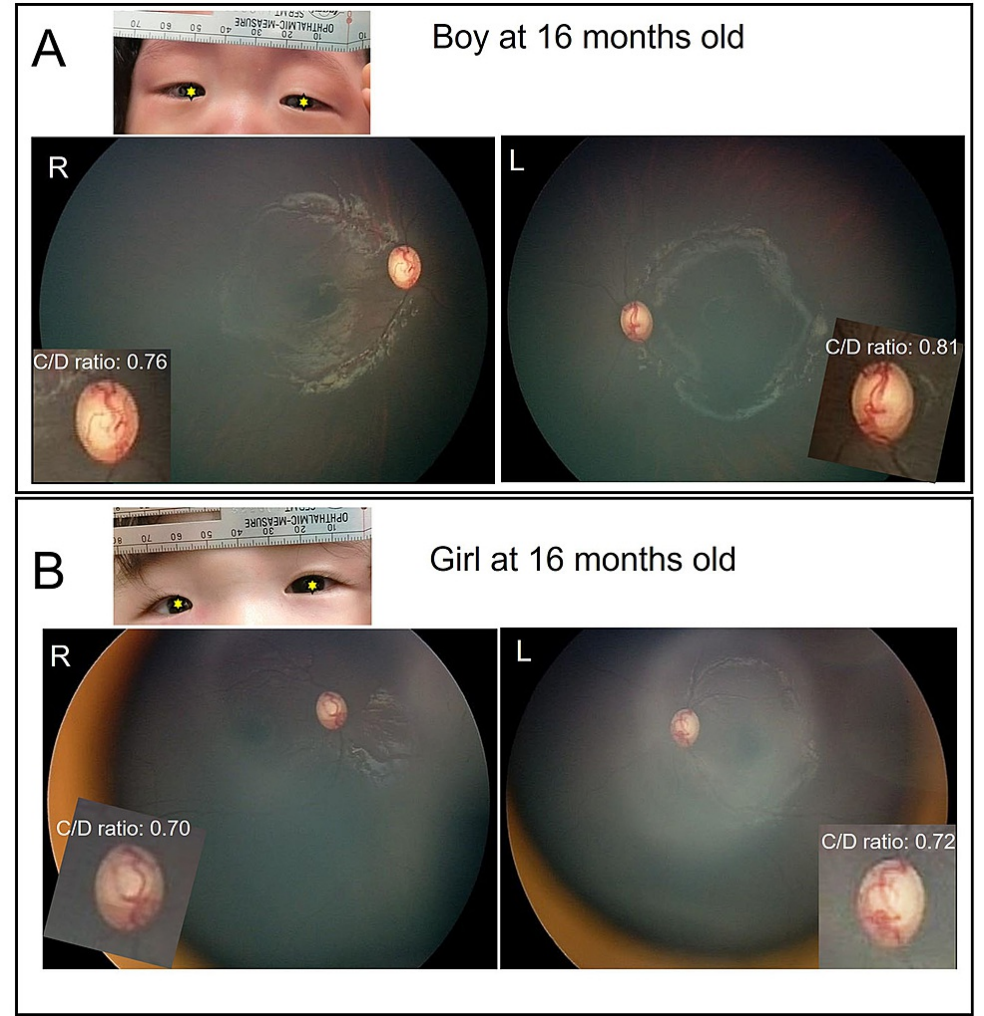
Color fundus photography and facial photographs at 16 months of age

The results of all the examinations at each visit are shown in Table 1. At 16 months of age, the babies’ horizontal corneal diameters did not change remarkably. The axial lengths and anterior chamber depths increased slightly. However, the spherical equivalent measured by Spot™ Vision Screener (Welch Allyn, New York, America) showed no apparent myopic shift. Additionally, the IOPs were stable at under 22 mmHg during the follow-up period. Fundus photography captured by Retcam at 16 months (Figure 2) showed no obvious enlargement of the C/D ratio in either child.

**Table 1:** Patients’ data during follow-up

	Boy		Girl	
Eye	Right eye	Left eye	Right eye	Left eye
C/D ratio				
Vertical C/D ratio at 8 months of age	0.77	0.81	0.86	0.75
Vertical C/D ratio at 16 months of age	0.76	0.81	0.7	0.72
Horizontal C/D ratio at 8 months of age	0.76	0.76	0.78	0.83
Horizontal C/D at 16 months of age	0.77	0.76	0.85	0.82
Axial length and anterior chamber depth (mm)				
Axial length at 8 months of age	19.91	19.52	18.57	18.81
Axial length at 16 months of age	20.82	20.9	19.97	19.73
ACD at 8 months of age	2.73	2.90	2.93	2.68
ACD at 16 months of age	2.98	3.16	3.32	3.22
Corneal diameter (mm)				
Horizontal corneal diameter at 8 months of age	10	10.5	10.5	10
Horizontal corneal diameter at 1 year of age	11	10	10	10
Horizontal corneal diameter at 16 months of age	11	11	10	11
Refraction (diopter)				
Spherical equivalent at 8 months of age	−2.5	−0.25	0	0.25
Spherical equivalent at 9.5 months of age	−1.25	−0.75	−0.25	−0.5
Spherical equivalent at 1 year of age	−1.25	−0.25	−1.75	−1
Spherical equivalent at 16 months of age	0.75	1.25	0	0.75
IOP (mmHg)				
IOP at 8 months of age	19.7	16.9	18.9	15.3
IOP at 8.5 months of age	22.1	21.2	21.6	22.1
IOP at 9.5 months of age	21.6	21.7	16	22.6
IOP at 1 year of age	18	18	10	11
IOP at 16 months of age	14	16	9	6

**Figure 3:**
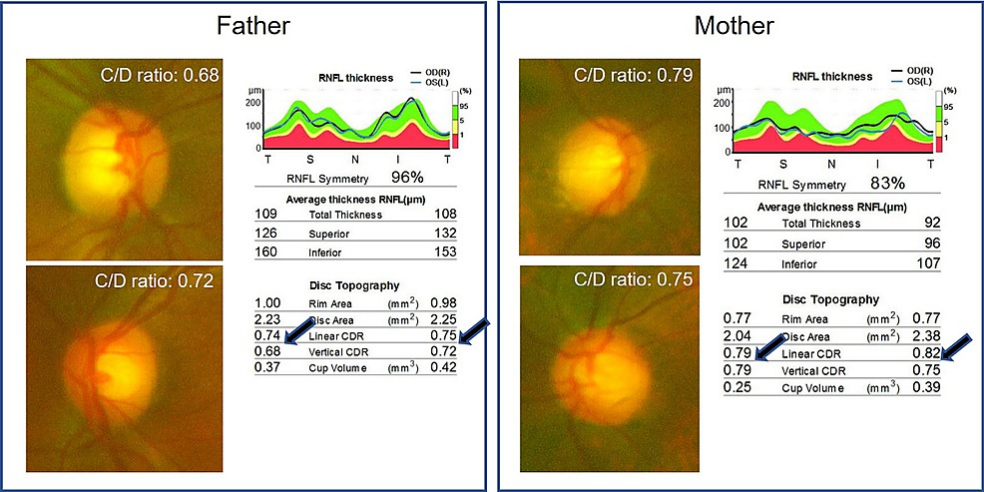
Optical coherence tomography (OCT) analysis in the parents

We evaluated the parents’ optic disks using optical coherence tomography (DRI OCT Triton, Topcon Corporation, Nagoya, Japan) to find out whether genetics factors were involved. As a result, OCT showed that both parents had large C/D ratios (0.68-0.79) (Figure 3).

## Discussion

Our cases showed no apparent ocular biometry changes and a normal IOP range during the follow-up period. Fortunately, signs such as corneal edema, enlargement of the eye with buphthalmos, and Haab striae due to high IOP were not found. According to a previous study into child glaucoma [2], a corneal diameter greater than 13 mm in a child of any age is abnormal, and corneal enlargement due to IOP occurs before three years of age. Additionally, the normal axial length at 15 months is between 20 and 22.6 mm (95% confidence interval) [2]; therefore, the data of our cases were within the normal ranges. Meanwhile, a C/D ratio of >0.3 in a Caucasian infant younger than one year and >0.5 in an older child can indicate glaucoma [2]. Only optic disk appearance looks unlike normal baby in our cases. We searched previous studies in PubMed using the keywords “preterm infant” and “cup disk ratio” or “large cupping disk” or “large cup disk” and found 14 papers published between 1978 and 2018 [3-16]. Six papers reported that the C/D ratio is larger in premature infants than in full-term infants [4,5,7,9,10,15]. Three papers suggested that the C/D ratio is similar in preterm babies and full-term babies [3,6,8]. Therefore, the idea that premature infants have larger cupping disks was predominant. Wikstrand’s report also supported that a low birth weight (SD score) and a low weight at week 32 (SD score) were associated with a larger area of the optic cup (r = −0.37, p＝ 0.0083, and r = −0.38, p = 0.0064, respectively) as well as with a smaller neuronal rim area of the optic nerve head (r = 0.35, p = 0.010, and r = 0.31, p = 0.023) [17].

However, genetic factors from the parents should be also considered. In our case, neither parent had a history of hereditary optic neuropathy. He et al. reported that the C/D ratio was 78.6% for genetic and 21.4% for unshared environmental effects [18]. Park et al. reported that a family history of large C/D ratio was a significant factor associated with a large vertical C/D ratio in children [19]. Han et al. reported that the narrow-sense heritability of the C/D ratio was 47%-48% [20]. Healey et al. reported that genetic factors explained 73%, 66%, and 34% of variations in optic disk, cup, and rim areas, respectively [21]. Environmental factors also seem to be important [18,21]. Overall, genetic factors are considered to be important for child optic disk shapes.

## Conclusions

We speculated that with lack of positive family history and the classical "triad" of PCG, the early birth with the low birth weights and the large cupping of the patients’ optic disks may have resulted in the optic disks' shapes in the two babies. Further careful long-term follow-up is needed to differentiate between normal physiological large cupping and PCG. Additionally, gonioscopy and visual field tests need to be conducted as the patients grow. Moreover, ocular morphological examination of the eye, IOP measurements, and investigation of the parents’ optical disks using fundus photography were useful in helping us differentiate between normal physiological large cupping and PCG in this case.
